# Effect of A-Cation Radius on the Structure, Luminescence, and Temperature Sensing of Double Perovskites A_2_MgWO_6_ Doped with Dy^3+^ (A = Ca, Sr, Ba)

**DOI:** 10.1021/acs.inorgchem.3c02798

**Published:** 2023-11-29

**Authors:** Thi Hong Quan Vu, Dagmara Stefańska, Przemysław Jacek Dereń

**Affiliations:** Włodzimierz Trzebiatowski Institute of Low Temperature and Structure Research, Polish Academy of Sciences, Okólna 2, Wrocław 50-422, Poland

## Abstract

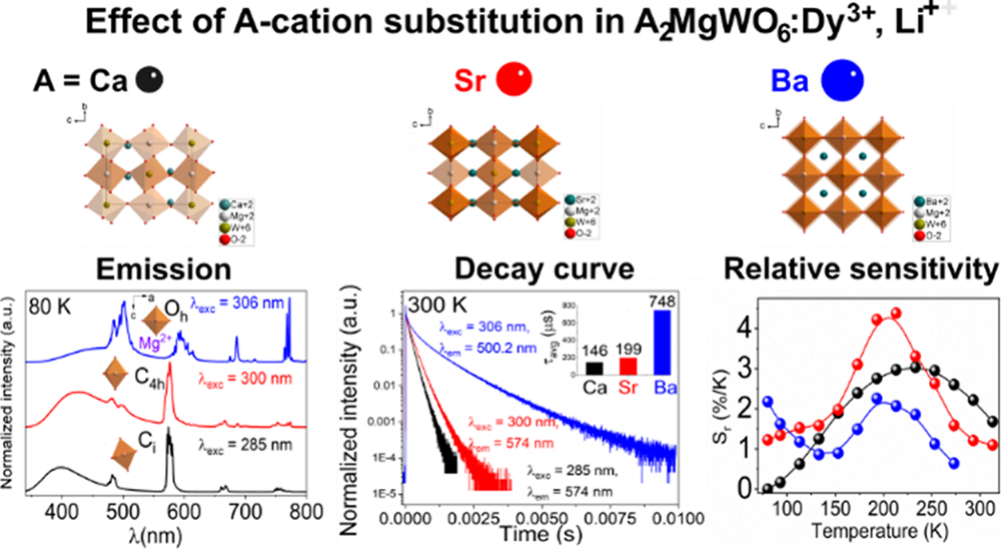

Herein, we report a case of A_2_MgWO_6_ (A = Ca, Sr, Ba) doped with 2%Dy^3+^, 2%Li^+^, in which the influences of the cation substitution are exhibited through the crystal structure, the charge transfer band (O^2–^–W^6+^), the emission spectrum, the color of the luminescence, and the luminescent decay time. The substitution of Ca^2+^ and Sr^2+^ ions for larger ions (Ba^2+^) led to the crystal structure alteration from cubic to monoclinic and tetragonal, respectively. These structure changes also lowered the crystallography symmetry site of Dy^3+^, tuned the color of the emitted light from the whitish to yellowish region, and caused a blue shift of the CTB. Furthermore, a significant decline in the lifetime of the ^4^F_9/2_ → ^6^H_13/2,15/2_ transitions was noticed, from 748, 199, to 146 μs corresponding to Ba, Sr, Ca sample owing to the reduction in the local symmetry of Dy^3+^. Moreover, the thermal sensing properties of 2%Dy^3+^-doped samples were investigated based on the fluorescence intensity ratio technique in the range of 80–325 K. Under 266 nm excitation wavelength, the maximum relative sensitivity of the investigated samples was remarkably enhanced from 2.26%/K, 3.04%/K, to 4.38%/K corresponding to Ba, Ca, and Sr samples, respectively. In addition to providing a comprehensive understanding of the effects of compositional modifications on the optical properties, the results also present a viable pathway to manipulate the temperature sensing performance.

## Abstract 2

The study provides a comprehensive understanding of the effects of cation substitution at the A-site in A_2_MgWO_6_:Dy^3+^,Li^+^ (A = Ca, Sr, Ba) double perovskites on the crystal structure, luminescent properties, emission decay time, and the relative sensitivity of the sample.

## 1 Introduction

In recent years, optical thermometry has become an intriguing research topic, as it offers ample advantages such as high accuracy, reliability, durability, and quick response compared to traditional sensors.^1^ In an attempt to enhance the sensitivity of optical thermometers, the effects of morphology,^2^ site-occupation preference,^3^ dopant concentration,^2^ band gap engineering,^4^ codopants,^5−9^ and synthesis methods^10^ on thermometric parameters have been investigated. Particularly, a structure change resulting from cation substitution has been demonstrated as an effective way to tune the temperature sensing performance.^11^

Double perovskite compounds (A_2–*x*_A_*x*_^′^BB^′^O_6_) are among the most intensely studied materials by virtue of their unique chemical and physical properties, as well as their diverse applications stemming from their structural flexibility. The crystal structure of double perovskites can be intentionally regulated by a cation substitution. To predict the crystal structure, tolerance factor (*t*) is applied using the modified Goldschmidt’s equation below^12,13^
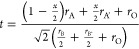
1where *r*_A_, *r*_A^′^_, *r*_B_, *r*_B^′^_, and *r*_O_ are the ionic radii of the corresponding A, A′, B, B′ cations, and the oxygen.

Many double perovskites have been reported so far, with the most common divalent cations at the A-site are Ca, Sr, and Ba.^14^ Among them, tungstate double perovskite materials A_2_MgWO_6_ have emerged as promising candidates for light-emitting diodes (LEDs)^15−17^ and temperature readout applications^2,10,17,18^ because of their high thermal stability, and broad absorption band in the UV range. To adjust the luminescent properties of materials, a common way is to alter the cation A.^19^ A series of Mn^4+^ activated A_2_MgWO_6_ (A = Ca, Sr, Ba) was investigated as a red component for white LEDs.^15^ Notably, Ba_2_MgWO_6_ (BMW) exhibits intense blue emission from the [WO_6_] host.^10^ Leveraging the simultaneous emission of [WO_6_] and the dopant, BMW-doped lanthanides have been widely investigated for white-light emission and self-reference optical thermometers.^17^ Furthermore, substituting Sr for Ba in BMW caused a blueshift in both excitation and emission spectra,^16^ along with a structural change from cubic to tetragonal.^11^ More importantly, a phase transition in Ba_(2_–_*x*)_Sr_*x*_MgWO_6_:Er,Yb resulted in an improvement in the maximum relative sensitivity of the thermometer, increasing from 0.8%/K to 0.9%/K, respectively.^11^ While Ca_2_MgWO_6_ (CMW) has been studied over the past few years, the main focus has been on ceramic fabrication^20,21^ and optical thermometry based on the up-conversion luminescence of Er^3+^ and Yb^3+22^. Surprisingly, the luminescent properties of rare-earth-ion-activated −Sr_2_MgWO_6_ (SMW) remain unknown.

Introducing an optical probe into the lattice is a facile method for identifying crystal structure changes in the emission spectrum. Dysprosium (Dy^3+^) is chosen as the probe because its luminescence is strongly affected by the site symmetry of the cation it replaces. When Dy^3+^ occupies a high symmetry site with an inversion center, the blue emission (^4^F_9/2_ → ^6^H_15/2_) usually dominates over other emission peaks. In contrast, the yellow emission (^4^F_9/2_ → ^6^H_13/2_) is the most intense.^23,24^ The yellow/blue (Y/B) ratio can be intentionally modulated by varying the chemical composition or the concentration of Dy^3+^ ions in perspective to obtain single-phase white-light-emitting phosphors.^25^ This Y/B ratio also provides information about the local environment around the Dy^3+^ ions: the higher the ratio of yellow-to-blue emission (≫1) often indicates a lower site symmetry of Dy^3+^.^24,25^

As highlighted above, the aim of this study is to verify the correlation between chemical modification and the structure, optical properties, and temperature sensing performance of a series of A_2_MgWO_6_:Dy^3+^,Li^+^ (A = Ca, Sr, Ba) double perovskites were obtained for the first time. All compounds were synthesized by using the coprecipitation method. It was noticed that when Ca, Sr, and Ba were successively situated at the A-site, the crystalline structure was modulated from monoclinic to tetragonal, and then to cubic. The consequences of the structural change on the emission characteristics and temperature-sensing capability of the compounds were discussed in detail.

## 2 Experimental Section

### 2.1 Materials and Synthesis

The precursors used for the syntheses include calcium nitrate Ca(NO_3_)_2_·4H_2_O, (Alfa Aesar, 99%), strontium acetate Sr(CH_3_COO)_2_·0.5H_2_O (Alfa Aesar, 99%), barium acetate Ba(CH_3_COO)_2_ (Alfa Aesar, 99%), magnesium acetate Mg(CH_3_COO)_2_·4H_2_O (Alfa Aesar, 99.95%), ammonium paratungstate (NH_4_)_10_H_2_(W_2_O_7_)_6_ (Sigma-Aldrich, 99.99%), dysprosium oxide Dy_2_O_3_ (Alfa Aesar, 99.99%), lithium carbonate Li_2_CO_3_ (Alfa Aesar, 99.998%), and nitric acid HNO_3_ (POCH, 65%). All of the precursors were used without any further purification.

Only the high-temperature solid-state reaction method was used for the syntheses of Ca_2_MgWO_6_^22,26^ and Sr_2_MgWO_6_.^27,28^ In this work, the powders of A_2_Mg_1.2–2*x*_Dy_*x*_Li_*x*_WO_6_ double perovskites (A = Ca, Sr, Ba; *x* = 0, 0.001, 0.005, 0.01, 0.02, 0.03, 0.05, 0.07) were synthesized by using the coprecipitation method.^16,29^ To compensate for the evaporation of Mg during high-temperature sintering, a 20% molar excess of Mg(CH_3_COO)_2_ was added to the synthesis. The introduction of Dy^3+^ ions to the Mg^2+^ site would cause lattice distortion due to the difference in ionic radii and charge mismatch. As a consequence, two types of defect structures can be considered: (1) 3Mg^2+^ = 2Dy^3+^ + V_Mg_, where V_Mg_ is the Mg^2+^ ion vacancy and (2) 2Mg^2+^ = Dy^3+^ + Li^+^. Therefore, lithium was applied as a charge compensator to maintain the charge balance and limit the defects. The amounts of Dy^3+^ and Li^+^ ions were calculated to respond with the Mg^2+^ ions, according to the chemical formula. The amount of precursors was calculated to achieve a total of 0.5 g of the final product.

Initially, an aqueous solution 1 containing tungstate ions, as well as a solution 2 comprising magnesium ions and cation A, were each dissolved in 20 mL of distilled water. Afterward, dysprosium oxide was dissolved in a nitric acid solution to create a dysprosium nitrate solution with a concentration of 1.67 × 10^–5^ mol of Dy^3+^ per ml. The stoichiometric amount of Dy^3+^ was then added to the solution containing Mg^2+^ and A^2+^. Following this, solution 1 was gently introduced into solution 2, with continuous stirring at 200 rpm at room temperature. The resulting precipitate was subjected to drying at 80 °C for 24 h and subsequently annealed twice in a corundum crucible under ambient conditions. The first sintering process occurred at 600 °C for 12 h for all samples, while the second step was carried out individually at 1000 °C for CMW, 1150 °C for SMW, and 1200 °C for BMW for 6 h with a heating rate of 3 °C per minute.

### 2.2 Characterization

The phase purity of the synthesized samples was confirmed through X-ray powder diffraction analysis. The measurements were conducted using the X’Pert PRO powder diffractometer (PANalytical, The Netherlands) equipped with a linear PIXcel detector and using CuKα radiation (λ = 1.54056 Å) in the 2θ range of 10°–90°. The XRD data were analyzed using the Le Bail refinement method^30^ implemented in the FullProf software package.^31^ During the analysis, the lattice parameter, instrumental zero-shifts, and full width at half-maximum (fwhm) parameters of the peaks of each diffractogram were fitted simultaneously. A typical pseudo-Voigt-type function was used to fit the Bragg peaks. For background correction of the XRD patterns, we used a six-coefficient polynomial function except for CMW. As CMW (host and the Dy doped) crystallizes in lower symmetry, a linear interpolated custom-chosen background line was used. The morphology was investigated based on the SEM images, which were obtained with a beam energy of 5 keV. The actual amount of lithium was verified by the inductively coupled plasma mass spectroscopy (ICP-MS ETHOS instrument, Italy). The excitation, emission spectra, and luminescent decay profiles were recorded by using an FLS1000 photoluminescence spectrometer (Edinburgh Instruments, UK) equipped with a xenon arc lamp (230–1000 nm), a visible PMT-980 detector, and a Tektronix MD3052 mixed domain oscilloscope 500 MHz (Beaverton, USA). Additionally, the temperature-dependent luminescent spectra were studied using the Hamamatsu photonic multichannel analyzer PMA-12 equipped with a BT-CCD linear image sensor (Hamamatsu Photonics K.K., Shizuoka, Japan) and a 266 nm diode laser. During the measurements, the samples’ temperature was controlled by a Linkam THMS 600 Heating/Freezing Stage (McRONE group, Westmont, USA).

## 3 Results and Discussion

### 3.1 Structure and Morphology

The XRD patterns of all compounds are shown in Figure S1 (Supporting Information file). CMW contains small impurity peaks at 18.7°, 28.9°, 31.7° (CaWO_4_) and 22.3° (Ca_3_WO_6_) (see Figure S1a). There are also some additional diffraction lines of Ba_2_WO_5_ (28°) and Ba_3_WO_6_ (29.4°) present in the BMW samples (Figure S1c). Only the samples containing Sr are free of impurity phases (Figure S1b). The amount of the impurity phases in the CMW and BMW samples was lower for the 2% concentration of Dy^3+^, Li^+^. Thus, the hosts and the samples doped with 2%Dy^3+^, 2%Li^+^ were chosen for further investigation.

**Figure 1:**
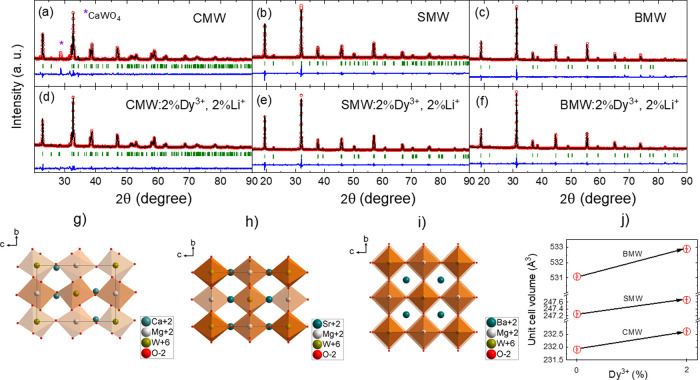
XRD of (a) CMW host, (b) SMW host, (c) BMW host, (d) CMW:2%Dy^3+^,2%Li^+^, (e) SMW:2%Dy^3+^,2%Li^+^, (f) BMW:2%Dy^3+^,2%Li^+^; the red circles and the black and blue lines correspond to the experimental data, fitted data, and the difference between the experimental and fitted data, respectively. The olive vertical bars correspond to the allowed Bragg positions by the space group. Visualization of crystal structures of (g) CMW, (h) SMW, and (i) BMW; (j) variations of the unit cell volumes of all studied samples.

A few studies on the crystal structure of CMW,^32^ SMW,^33,34^ and BMW^35^ have been published previously. BMW was reported as cubic^15,16^ while CMW crystallized in monoclinic,^15,22,26,32,36−40^ orthorhombic^41^ or cubic.^20^ SMW showed stability of the tetragonal phase up to 40 GPa^33^ and revealed a continuous phase transition at 570 K, changing the symmetry from tetragonal to cubic.^34^ Therefore, to obtain the accurate crystal structure of the synthesized samples, the Le Bail refinement method was applied.^30^ The refined XRD patterns of the compounds CMW, SMW, and BMW (both host and 2%Dy^3+^2%Li^+^ substituted) are shown in Figure 1a–c. The refined lattice parameters, unit cell volumes, and statistical uncertainties while fitting are summarized in Table S1. The results show that BMW exhibits a cubic phase with the space group of *Fm*3̅*m* (#225) while SMW can be indexed into the tetragonal *I*4/*m* (#87), and CMW belongs to the monoclinic *P*2_1_/*n* (#14).

The investigated compounds are typical examples of B-site-ordered perovskites with the general formula A_2_BB′O_6_, where A cations are coordinated with 12 oxygen atoms (CN = 12), and both B and B′ are in 6-coordination (CN = 6). The octahedra formed by B and B′ ions share oxygen at the lattice corners. From Table 1, the crystal structure can be expected to change from a cubic structure (BMW, *t* = 1.03), to a tetragonal structure (SMW, *t* = 0.972), and finally to a monoclinic structure (CMW, *t* = 0.938). This prediction is in good agreement with the refined data presented in Table S1. A reduction in the value of *t* exhibits a pronounced distortion of the structure when Ba^2+^ ions are replaced by Ca^2+^ and Sr^2+^ ions, showing the highly distorted octahedra in CMW and SMW (see Figure 1g,h). As shown in Figure 1j, the unit cell volume of all doped samples expanded, indicating the successful substitution of Mg^2+^ ions (0.72 Å) by Dy^3+^ ions (0.912 Å). All ionic radii data were obtained from the study of Shannon.^42^

**Table 1:** Behavior of the Crystal Structure as a Function of the Tolerance Factor^1,4^

*t* value	*t* < 0.97	0.97 ≤ *t* < 1	1 ≤ *t* < 1.05	*t* ≥ 1.05
structure	monoclinic, orthorhombic	tetragonal	cubic	hexagonal

The morphological properties and grain sizes of the samples doped with 2%Dy^3+^ and 2%Li^+^ are clearly shown in Figure S2. Overall, all samples exhibited a heterogeneous surface, and individual grains were not observed. The SEM images illustrate that the crystallites strongly agglomerated and stacked together, forming larger particles with irregular shapes, particularly noticeable in the case of CMW:2%Dy^3+^,2%Li^+^. The boundaries between these crystallites were not identifiable. The largest crystallites could be recognized, reaching sizes of up to 400 nm for SMW:2%Dy^3+^,2%Li^+^ and BMW:2%Dy^3+^,2%Li^+^, while in the case of CMW2%Dy^3+^,2%Li^+^, they were found to be several μm in size. Indeed, the coprecipitation method helps to reduce the crystallite sizes of the synthesized samples as compared to those prepared by the traditional solid-state method. For example, CMW:Bi^40,41^ prepared by the solid-state method had larger grain sizes ranging from 6 to 60 μm. Similarly, BMW:Eu, prepared using the mechanochemical method,^2^ were also found to have sizes of approximately 6 μm. Due to the high-temperature sintering, the actual weight percentage of lithium ions was found to be half of the nominal amount (see Table S2).

### 3.2 Excitation Spectra

**Figure 2:**
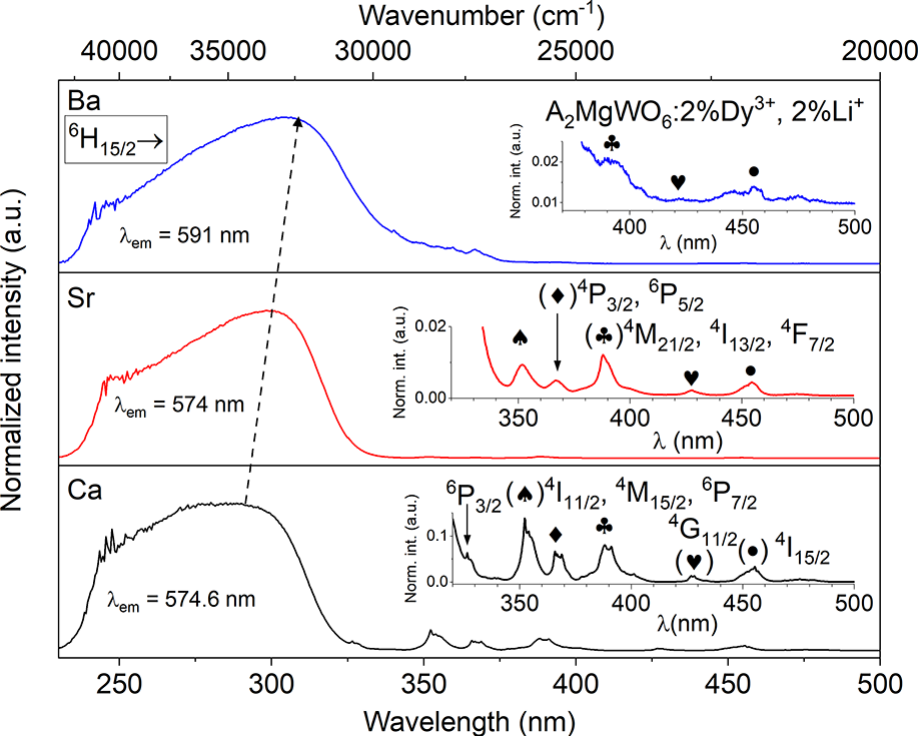
Excitation spectra (300 K) of A_2_MgWO_6_:2%Dy^3+^,2%Li^+^ where A = Ca (black), Sr (red), and Ba (blue) monitored at the ^4^F_9/2_ → ^6^H_13/2_ transition. Please note that free ozon lamp emits from 230 nm.

The room temperature excitation spectra of the samples doped with 2%Dy^3+^, 2%Li^+^ monitored at ^4^F_9/2_ → ^6^H_13/2_transition, are presented in Figure 2. Since our xenon arc lamp emits from 230 nm, the initial signal is weak and unstable, leading to unavoidable noise at around 242 nm. Each spectrum is composed of a broadband from 230 to 350 nm, which is ascribed to the charge transfer band (CTB) of the O^2–^–W^6+^. Besides, the f–f transitions of Dy^3+^ from the ^6^H_15/2_ ground state to the excited states are observed. The excitation peaks in the spectrum of CMW:2%Dy^3+^,2%Li^+^ are distinctly separated and more recognizable than those observed for samples containing Sr and Ba. These excitation peaks are attributed to the following transitions of ^6^H_15/2_ → ^6^P_3/2_ at 326 nm, ^6^H_15/2_ → ^4^I_11/2_, ^4^M_15/2_, ^6^P_7/2_ with the maximum centered at 352 nm, ^6^H_15/2_ → ^4^P_3/2_, ^6^P_5/2_ at 366 nm, ^6^H_15/2_ → ^4^ M_21/2_, ^4^I_13/2_, ^4^F_7/2_ at 388 nm, ^6^H_15/2_ → ^4^G_11/2_ at 427 nm, and ^6^H_15/2_ → ^4^I_15/2_ at 455 nm, respectively.^43^

The CTB was deconvoluted into four Gauss components on the spectra of all samples (Figure S3 and Table S3). From previous studies,^44,45^ the first and second components with higher energy can be assigned to the regular WO_6_ groups (REG), whereas the third and fourth components with lower energy represent the irregular ones (IRREG, when W^6+^ enters Mg^2+^ sites). The CTB’s maximum in Ca, Sr, and Ba samples undergoes a redshift from 285, 300, and 306 nm (black dashed arrow, Figure 2), respectively. This redshift is caused by a significant difference in the ratio between the integrated intensity of the excitation bands associated with the regular and irregular WO_6_ groups (see Table S3). For the Ca, Sr, and Ba samples, this ratio decreases significantly from 3, 2.2, to 0.9, respectively.

### 3.3 Emission Spectra

**Figure 3:**
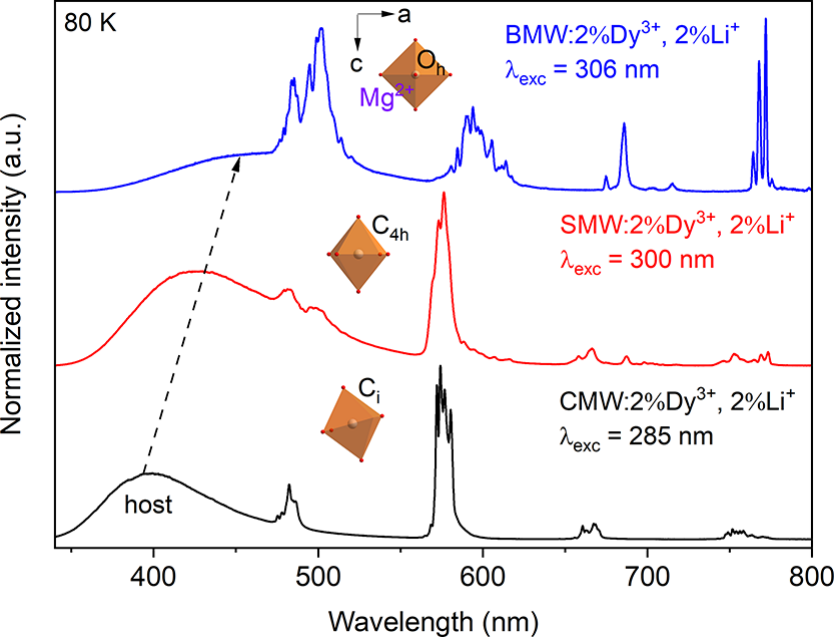
80 K emission spectra of A_2_MgWO_6_:2%Dy^3+^,2%Li^+^ where A = Ca (black), Sr (red), and Ba (blue) excited at 285, 300, and 306 nm, respectively. Inset: illustration of the change in the site symmetry of Mg^2+^ in octahedra in the given compounds.

**Figure 4:**
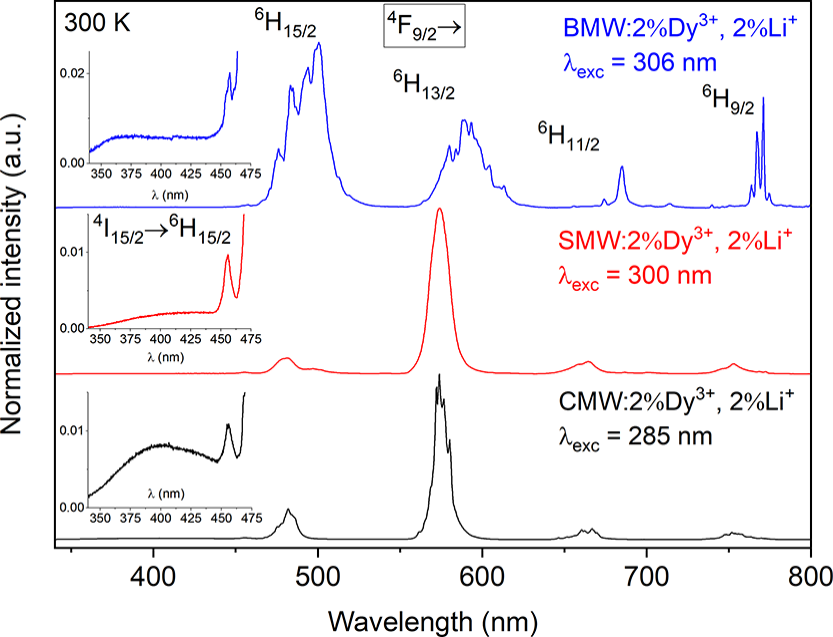
Emission spectra (300 K) of A_2_MgWO_6_:2%Dy^3+^,2%Li^+^ where A = Ca (black), Sr (red), and Ba (blue) excited at 285, 300, and 306 nm.

Figures 3 and 4 present the 80 and 300 K emission spectra of A_2_MgWO_6_:2%Dy^3+^,2%Li^+^ compounds upon the excitation at 285, 300, and 306 nm, respectively. The spectra consist of a broad and intense emission band from the tungstate host (345–500 nm), along with typical f–f transitions of Dy^3+^ (Figure 3). The peaks observed at 481, 574, 665, and 754 nm correspond to the transitions from the ^4^F_9/2_ level to ^6^H_15/2_, ^6^H_13/2_, ^6^H_11/2_, and ^6^H_9/2_ levels (Figure 4). At 300 K, a small and sharp line assigned to the ^4^I_15/2_ → ^6^H_15/2_ transition is observed at 456 nm (Figure 4).

As the temperature increases, two features can be noted: a decrease in the host emission intensity and a broadening of the emission peaks of Dy^3+^. The decrease in the host emission intensity is attributed to the accelerated energy transfer process from WO_6_ to Dy^3+^ ions at higher temperatures. Meanwhile, the broadening of the emission peaks of Dy^3+^ is primarily a result of increased phonon vibrations, which become more pronounced with rising temperature. At 80 K, the peaks are more structured and distinguishable compared to those observed at 300 K (especially for SMW).

Similar to the CTB, the maximum of the host emission also shifts toward longer wavelengths, from 401 nm via 433 to 444 nm (as indicated by the dashed arrow in Figure 3). This phenomenon relates to variations in proportions of the integrated emission intensity between the regular and irregular WO_6_ groups within each compound (refer to Figure S4 and Table S4). In the case of BMW:2%Dy^3+^,2%Li^+^, emission from the irregular WO_6_ groups constitutes a vast majority of the host’s emission, accounting for over 80%. Conversely, for Sr and Ca samples, this proportion is 0.76 and 1.36, respectively (Table S4).

**Figure 5:**
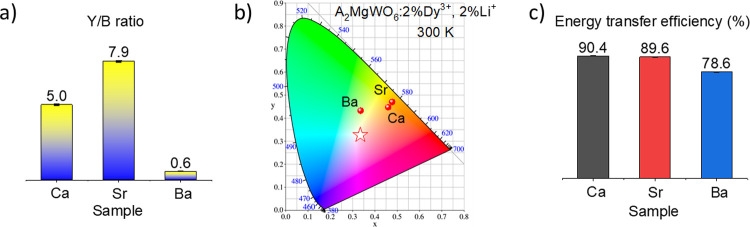
(a) Y/B ratio; (b) CIE 1931 chromaticity diagram; and (c) energy transfer efficiency (%) of A_2_MgWO_6_:2%Dy^3+^,2%Li^+^ where A = Ca (black), Sr (red), and Ba (blue).

Going from Ca, via Sr to Ba sample, the symmetric site of Mg^2+^ (where Dy^3+^ is occupied) alters from low symmetry (*C*_i_, and *C*_4h_), to high symmetry (*O*_h_), respectively (see the inset in Figure 3). This change in the site symmetry of Dy^3+^ has a significant effect on the emission spectra. In the emission spectra, the yellow emission peak (^4^F_9/2_ → ^6^H_13/2_) remains in the same position at 574 nm for the Ca and Sr phosphors. However, for the Ba compound, there is a clear displacement toward the red region (at 591 nm) because Dy^3+^ occupies a high symmetry site (*O*_h_) with an inversion center, where electric dipole transitions are entirely forbidden according to the SLJ selection rules. Consequently, the spectrum of BMW:2%Dy^3+^,2%Li^+^ exhibits vibronic lines, similar to the cases of Ba_2_CaWO_6_:Dy,^23^ Ba_2_ZnWO_6_:Dy.^46^ In these cases, the most prominent emission in the spectrum is the blue emission of the ^4^F_9/2_ → ^6^H_15/2_ transition. Conversely, when Dy^3+^ is located in a nonsymmetric site in the lattice, the predominant emission is the yellow emission of the ^4^F_9/2_ → ^6^H_13/2_ transition, as reported in various other materials.^47−51^ This phenomenon evidently influences the Y/B intensity ratio (Figure 5a) and the color of the emitted light (Figure 5b).

The Commission Internationale de l’Eclairage (CIE) 1913 chromaticity coordinates in Figure 5b illustrate the influence of the modification of the A-cation on the emitted light. The color of the luminescence shifts toward the yellow region with Y/B ≫ 1. Specifically, SMW and CMW samples doped with 2%Dy^3+^ and 2%Li^+^ show intense yellowish-orange luminescence with coordinates of (0.48, 0.47) and (0.46, 0.45), respectively. While BMW:2%Dy^3+^,2%Li^+^ exhibits greenish-yellow emission (0.33, 0.44), which is far from ideal white light (0.33, 0.33) (marked as a star). These results are comparable with other Dy^3+^-doped phosphors, such as BMW (0.28, 0.31),^52^ BCW (0.3, 0.37),^23^ Ca_3_WO_6_ (0.35, 0.37),^50^ CaWO_4_ (0.31, 0.31),^49^ Y_2_WO_6_ (0.32, 0.34),^51^ etc.

The 300 K emission spectra of the hosts and samples doped with 2%Dy^3+^, 2%Li^+^ are presented in Figure S5. The energy transfer efficiency (η_ET_) was estimated using the formula below:
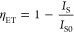
2where *I*_S_, *I*_S0_ is the integrated intensity of the sample containing Dy^3+^ and the host.

At 300 K, the most efficient energy transfer (90.4%) was found for CMW:2%Dy^3+^,2%Li^+^, followed by SMW:2%Dy^3+^,2%Li^+^ (89.6%), and BMW:2%Dy^3+^,2%Li^+^ (78.6%) (Figure 5c). The reader is also encouraged to look at the excitation spectra (Figure S3), paying attention to the excitation bands. It was demonstrated that the energy transfer from the WO_6_ to the activator (Dy^3+^ ions) occurs exclusively from the regular WO_6_ group.^2,10^ The energy transfer from WO_6_ to Dy^3+^ is more efficient if the major contribution to the excitation bands comes from the regular WO_6_ groups. For the Ba-compound, the components associated with the irregular WO_6_ group prevail over the regular ones, in contrast to the Ca-sample (see Table S3). Therefore, the energy transfer efficiency is the lowest in the BMW sample and the highest in the CMW sample.

**Figure 6:**
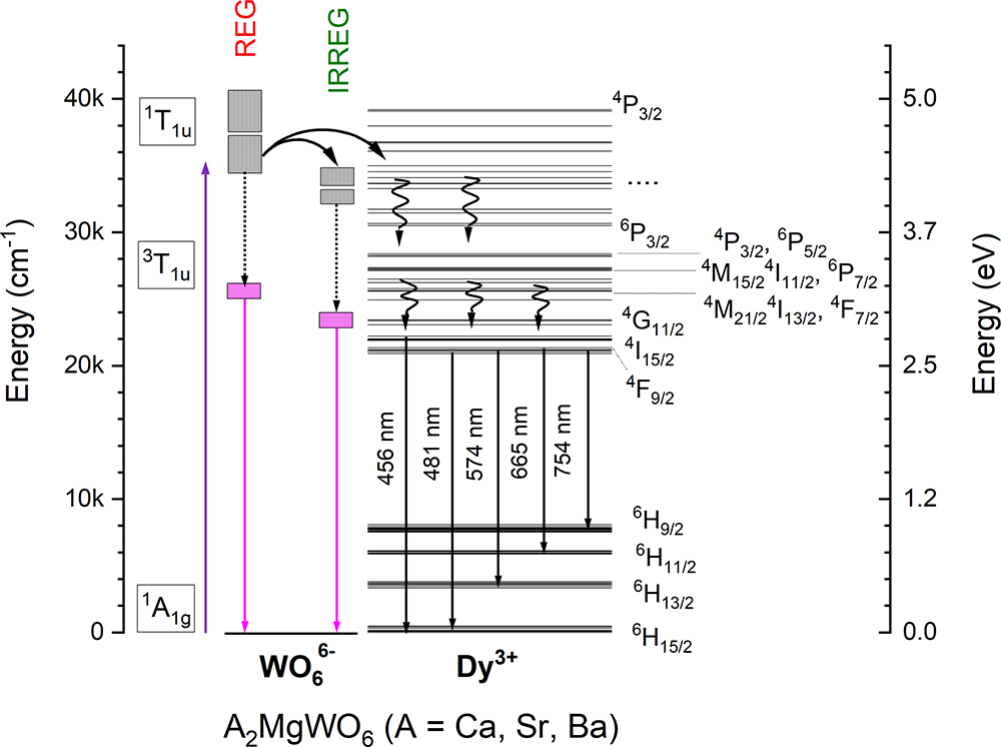
Energy scheme of regular and irregular WO_6_ groups, Dy^3+^ and the energy transfer processes, emissions, and nonradiative transitions occurring in A_2_MgWO_6_:2%Dy^3+^,2%Li^+^ where A = Ca, Sr, Ba. Black dot lines and wavy lines represent the nonradiative transitions, while the black solid curves and solid straight lines represent the radiative ones. The thickness of the ^1^T_1u_ level depends on the weight ratio among excitation bands.

To understand the energy transfer process from the WO_6_ group to Dy^3+^ occurring in A_2_MgWO_6_:Dy^3+^,Li^+^ compounds, a schematic energy diagram is constructed based on the excitation spectra and the emission spectra (Figure 6). The two excited ^1^T_1u_ levels were determined from the excitation bands of WO_6_ groups.^53^ The emissions from the ^3^T_1u_ level to the ^1^A_1g_ level are a result of two types of tungstate groups.

First, electrons are pumped into the CTB of the WO_6_ group. Subsequently, the excitation energy is transferred from the regular WO_6_ group to the irregular WO_6_ group and to Dy^3+^ through a resonance process (black solid curves). After that, the high energy excited levels of Dy^3+^ relax nonradiatively to the ^4^F_9/2_ and ^4^I_15/2_ levels by multiphonon relaxation. Finally, the emissions from ^4^F_9/2_ to ^6^H_15/2, 13/2, 11/2, and 9/2_ and ^4^I_15/2_ to ^6^H_15/2_ were observed. Besides, emission from the regular and irregular WO_6_ group were obtained.

### 3.4 Emission Decay Profiles

**Figure 7:**
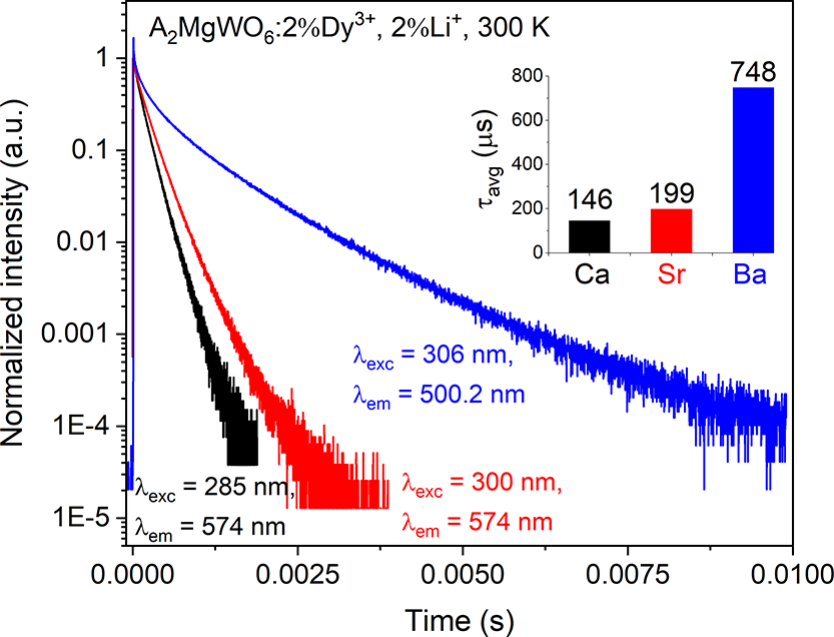
Luminescence decay curves (300 K) of A_2_MgWO_6_:2%Dy^3+^,2%Li^+^ where A = Ca (black), Sr (red), and Ba (blue). Inset: average lifetimes of the investigated samples.

The 300 K luminescent decay time of the ^4^F_9/2_ → ^6^H_13/2_ transition of Dy^3+^ ions in different hosts was investigated upon excitation at 285, 300, and 306 nm, monitoring at the strongest emission peak of each sample (Figure 7). The decay curves of all samples were not exponential due to the nonradiative process, therefore, the average decay times were estimated by the integral method:^43^
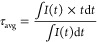
3

It can be seen that the average decay time of the ^4^F_9/2_ → ^6^H_13/2_ transition was lengthened, regarding the Ca, Sr, and Ba samples. When Dy^3+^ions were situated in a high symmetry site with an inversion center in the BMW lattice, the electric dipole transitions are completely forbidden according to the SLJ selection rules. Therefore, the longest average lifetime (748 μs) was obtained for the BMW lattice. In contrast, the average lifetimes of CMW:2%Dy^3+^,2%Li^+^ and SMW:2%Dy^3+^,2%Li^+^ are much shorter (146 and 199 μs, respectively) since Dy^3+^ is located in the low symmetry site (the electric dipole transitions are allowed). Such a very long lifetime was also obtained for Dy^3+^-activated phosphors with centrosymmetric sites, such as 450 μs for BMW:Dy (solid-state method),^52^ and 546 μs for Ba_2_CaWO_6_:Dy.^23^

### 3.5 Temperature Sensing Performance

**Figure 8:**
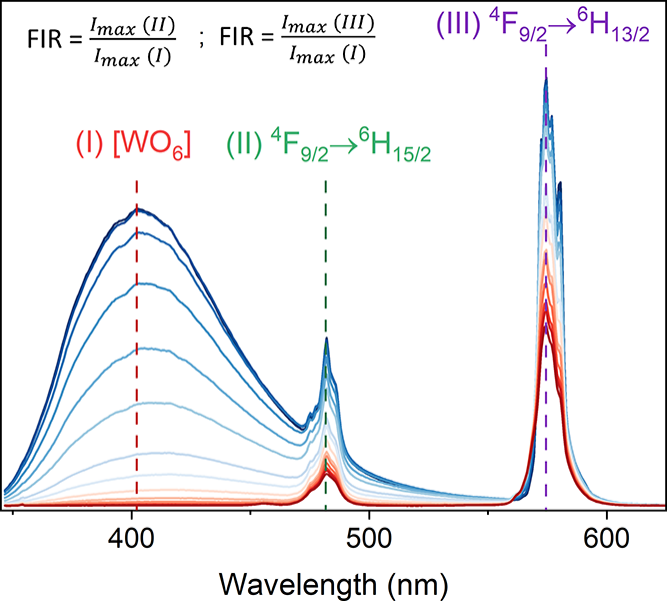
Schematic representation of the approach for temperature determination.

The significant dependency of luminescence intensity on temperature is a fascinating characteristic that can be harnessed for temperature readout, especially noncontact thermometry, utilizing the ratiometric luminescence of the host and dopant ions, such as [Ti_2_O_7_] and Eu^3+^,^54^ VO_4_^3–^ and Eu^3+^,^55^ [WO_6_] and Ho^3+^,^56^ Eu^3+^,^10^ Nd^3+^,^18^ Er^3+^.^17^ In this study, we propose an approach to design a thermometer based on the relationship between the [WO_6_] host emission (I) and the emission of ^4^F_9/2_ → ^6^H_15/2_ (II), and ^4^F_9/2_ → ^6^H_13/2_ (III) (see Figure 8). The fluorescence intensity ratio (FIR) parameter was modified due to the substantial overlap between channels (I) and (II), becoming the ratio of the maximum of channels (II) and (III) to the maximum of channel (I). Table 2 presents a summary of FIRs with their respective considered channels.

**Table 2:** Summary of FIRs in Each Host System

samples	FIR	
	channel III and I	channel II and I
CMW:2%Dy^3+^,2%Li^+^		
SMW:2%Dy^3+^,2%Li^+^		
BMW:2%Dy^3+^,2%Li^+^		

**Figure 9:**
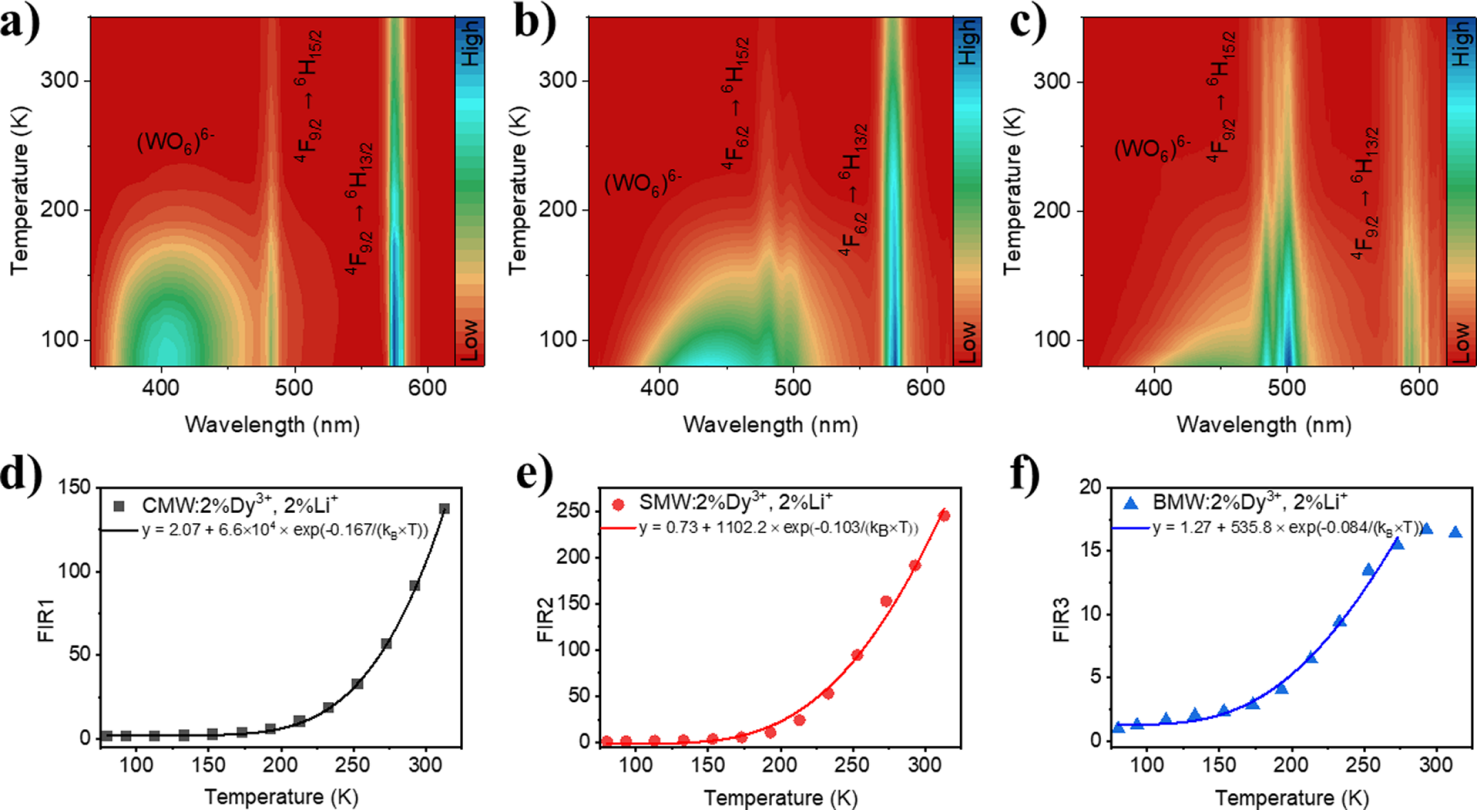
(a–c) Thermal evolution of the emission intensity (λ_exc_ = 266 nm) of A_2_MgWO_6_:2%Dy^3+^,2%Li^+^ where A = Ca, Sr, and Ba and (d–f) FIR_1,2,3_ as a function of temperature.

Since the emission of [WO_6_] was virtually quenched at room temperature (Figures S6–S8), the temperature-dependent emission intensity was discussed within the range of 80–325 K (Figure 9). Generally, an increase in the temperature results in a corresponding increase in the value of FIR (Figures 9d–f and S9). However, for the sample containing Ba, the increase in the FIR is observed up to 275 K, beyond which it shows a downward trend. The highest values of FIR are obtained for SMW:2%Dy^3+^,2%Li^+^ among the pairs originating from channels (III) and (I). Regarding the pairs of (II) and (I), the FIR values are similar for all samples.

The temperature-dependent FIR shows a Boltzmann-like behavior, therefore, it can be fitted to the below equation:^57^
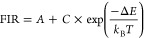
4where *k*_B_ is the Boltzmann constant, *T* is the temperature, and Δ*E* is the energy difference between the two involved levels.

**Figure 10:**
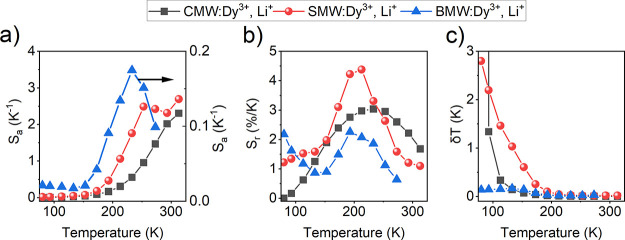
(a) Absolute sensitivity *S*_a*_*1,2,3_ (b) relative sensitivity *S*_r_1,2,3_ (c) temperature uncertainty δ*T*_1,2,3_ versus temperature.

To clarify the luminescent thermometry performance, the absolute (*S*_a_, K^–1^) and relative (*S*_r_, %/K) sensitivities, given by each channel, along with the corresponding temperature uncertainties δ*T* (K) were calculated using the following equations^58^ (Figure 10).
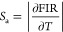
5
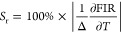
6
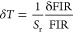
7where δFIR represents the change of FIR at temperature change δ*T*. δFIR/FIR is the relative uncertainty of the measurement.

In general, an upward tendency of the absolute sensitivities as a function of the temperature is observed for all samples. Particularly, for the Ba-sample, the absolute sensitivities initially increase with rising temperature up to 225 K, but then decrease significantly. Furthermore, the sample containing Ba is the most strongly affected by temperature change, as it possesses the highest values of *S*_a_ among all the samples (Figure 10a). As for the SMW:2%Dy^3+^,2%Li^+^ sample, it exhibits a steady stage from 250–300 K after an initial rise, followed by a sudden surge. On the other hand, CMW:2%Dy^3+^,2%Li^+^ displays a steep rise throughout the investigated temperature range (Figures 10a and S10a).

Similarly, the relative sensitivities *S*_r_ also increase with temperature. However, unlike *S*_a_, *S*_r_ allows us to compare the sensitivity performance of different materials, regardless of their nature.^58^Figure 10b presents bell-like relative sensitivity curves, with the exception of BMW:2%Dy^3+^,2%Li^+^, which shows a spine shape. The maximum value of *S*_r_, reaching 4.38%/K, is achieved at 213 K for SMW:2%Dy^3+^,2%Li^+^ to the best of our knowledge, making it the highest reported for the low-temperature range among luminescent Dy^3+^-based thermometers (Table 3). It is followed by a value of 3.04%/K at 233 K for CMW:2%Dy^3+^,2%Li^+^, while the lowest one of 2.26%/K is found at 193 K for BMW:2%Dy^3+^,2%Li^+^. Regarding channels (II) and (I), the greatest *S*_r_ is found to be 3%/K (at 253 K), 2.63%/K (at 213 K), and 2.03%/K (at 193 K) corresponding to the samples containing Sr, Ca, and Ba, respectively.

**Table 3:** Maximum Relative Sensitivities *S*_r,m_ at the Given Temperature *T* and Temperature Uncertainty δ*T* of Dy^3+^-Activated Optical Thermometers

material	temperature range (K)	*S*_r,m_ (%K^–1^)	*T*_m_ (K)	δ*T*_m_ (K)	ref
MgNb_2_O_6_:Pr,Dy	300–540	7.61	300	0.0045	(59)
Sr_2_MgWO_6_:Dy	80–313	4.38	213	0.04	this study
mixture of Li_2_TiO_3_:Mn and Y_2_O_3_:Dy	273–373	4.34	288		(60)
Ca_2_MgWO_6_:Dy	80–313	3.04	233	0.005	this study
Ba_2_MgWO_6_:Dy	80–273	2.26	193	0.03	this study
YAG:Dy,Cr	200–700	2.2	200		(61)
La_2_MgTiO_6_:Pr,Dy	300–550	2.1	550		(5)
BaYF_5_:Dy	293–773	2	293		(62)
YNbO_4_:Dy	293–773	1.97	300		(63)
SrWO_4_:Eu,Dy	11–592	1.71	335		(64)
YAG:Ti,Dy	123–523	1.6	373		(65)
GdPO_4_:Dy	290–539	1.55	290		(66)
GdAl_2_(BO_3_)_4_:Dy,Eu	300–475	1.37	475		(67)
Li_3_Y_3_Te_2_O_12_:Dy	80–300	1.2	80		(68)
Sc_2_W_3_O_12_:Dy	298–575	0.71	375		(69)

The δ*T* refers to the smallest change in temperature that the measurement can detect. This parameter strongly depends on the actual temperature of the sample under investigation and the experimental setup.^58^ The calculated δ*T* values at various temperatures are presented in Figure 10c. As the temperature increases, the δ*T* of the Ba-based sample stabilizes between 0.03 and 0.14 K, indicating excellent thermal resolution. However, for the Ca-, Sr-based samples, when the temperature falls below 100 K, the value of δ*T* surpasses 1 K. A similar phenomenon occurs only for the Ca-sample in the case of channel I and II.

For further investigation, the reliability of the thermal sensing performance was verified by assessing the repeatability (*R*%). The impact of heating and cooling processes on the optical properties of the samples is illustrated in Figure S11. The high value of *R*, above 99%, indicates the high reproducibility of the synthesized phosphors.

From Ca via Sr to Ba, the Sr-based sample exhibited the highest thermal sensing ability. The obtained maximum *S*_r_ values for A_2_MgWO_6_:2%Dy^3+^,2%Li^+^ (A = Ca, Sr, Ba) are higher than most of the optical thermometers collated in Table 3, which indicates that these materials are potential candidates for temperature detection applications in the low-temperature region.

## 4 Conclusions

A_2_Mg_1–2*x*_WO_6_:*x*Dy^3+^,*x*Li^+^ (*x* = 0, 0.02, and A = Ca, Sr, Ba) double perovskites were successfully synthesized by using the coprecipitation method for the first time. With an increase in the A-ionic radius (Ca < Sr < Ba), the crystal structure of A_2_MgWO_6_ changes successively from monoclinic to tetragonal and then to cubic, respectively. Besides, the color of emission changes from bright yellow (with the dominance of the ^4^F_9/2_→ ^6^H_13/2_ transition) to blue (mainly the ^4^F_9/2_→ ^6^H_15/2_) due to the alteration in site symmetry of Dy^3+^ in the structure, from *C*_1_*, C*_4h_ to *O*_h_, respectively. Additionally, a redshift of the host emission and the CTB of O^2–^–W^6+^ was observed due to the difference in the proportions of integrated intensity between the regular and irregular WO_6_ groups present in each sample. The longest emission decay time (748 μs) of Dy^3+^ in BMW is due to the location of Dy^3+^ in the center of inversion. The temperature sensing ability of all samples was verified by using the fluorescence intensity ratio of the ^4^F_9/2_→ ^6^H_15/2_, ^4^F_9/2_→ ^6^H_13/2_, with the host in the temperature range from 80 to 325 K. Among the investigated samples, the Sr_2_MgWO_6_:2%Dy^3+^,2%Li^+^ is found to be more sensitive to temperature with the highest *S*_r_ of 4.38%K^–1^. These results reveal the potential application of the studied compounds for optical thermometers in the low-temperature range.

## Supporting Information Available

XRD patterns of A_2_Mg_1–2*x*_WO_6_:*x*Dy^3+^,*x*Li^+^ (A = Ca, Sr, Ba; *x* = 0, 0.001, 0.005, 0.01, 0.02, 0.03, 0.05, 0.07); refined lattice parameters and unit cell volumes; SEM images; ICP-MS result of lithium; Gauss fitting of the 300 K excitation spectra; Gauss fitting of the 80 K emission spectra; 300 K emission spectra of A_2_Mg_1–2*x*_WO_6_:*x*Dy^3+^,*x*Li^+^ (A = Ca, Sr, Ba; *x* = 0, 0.02); temperature-dependent emission spectra; fluorescence intensity ratios; absolute sensitivities, relative sensitivities, temperature uncertainties; and repeatability (PDF) are available at 10.5281/zenodo.8211120.

## Notes

The data presented in this study will be made available on Zenodo with the DOI: 10.5281/zenodo.8211120 upon acceptance by the journal.

## Abbreviations

CMW: Ca_2_MgWO_6_
CMW: 2%Dy^3+^, 2%Li^+^ Ca_2_MgWO_6_:2%Dy^3+^,2%Li^+^
SMW: Sr_2_MgWO_6_
SMW: 2%Dy^3+^, 2%Li^+^ Sr_2_MgWO_6_:2%Dy^3+^,2%Li^+^
BMW: Ba_2_MgWO_6_
BMW: 2%Dy^3+^, 2%Li^+^ Ba_2_MgWO_6_:2%Dy^3+^,2%Li^+^
XRD: X-ray powder diffraction
CTB: charge transfer band
