# Supplementary material for: Effect of A-Cation Radius on the Structure, Luminescence, and Temperature Sensing of Double Perovskites A2MgWO6 Doped with Dy3+ (A = Ca, Sr, Ba)
Source: Inorg Chem. 2023 Nov 29;62(49):20020–9. doi: 10.1021/acs.inorgchem.3c02798 (PMC10716894; doi:10.1021/acs.inorgchem.3c02798)
Supplement: Supplementary file 1 — ic3c02798_si_001.pdf [file ic3c02798_si_001.pdf]

**Supporting information for**

# Effect of A-cation radius on the structure, luminescence, and temperature sensing of double perovskites $A_2MgWO_6$ doped with $Dy^{3+}$ (A = Ca, Sr, Ba)

Thi Hong Quan Vu*, Dagmara Stefańska, Przemysław Jacek Dereń

*Włodzimierz Trzebiatowski Institute of Low Temperature and Structure Research, Polish Academy of Sciences*

*Okólna 2, 50-422 Wrocław, Poland*

**Corresponding author: q.vu@intibs.pl*

## Crystallographic Data (Powder)

**Source:** The measurements were conducted using the X'Pert PRO powder diffractometer (PANalitycal, The Netherlands) equipped with a linear PIXcel detector and using CuKα radiation ($\lambda$ = 1.54056 Å) in the 2θ range of 10° to 90°.

a)

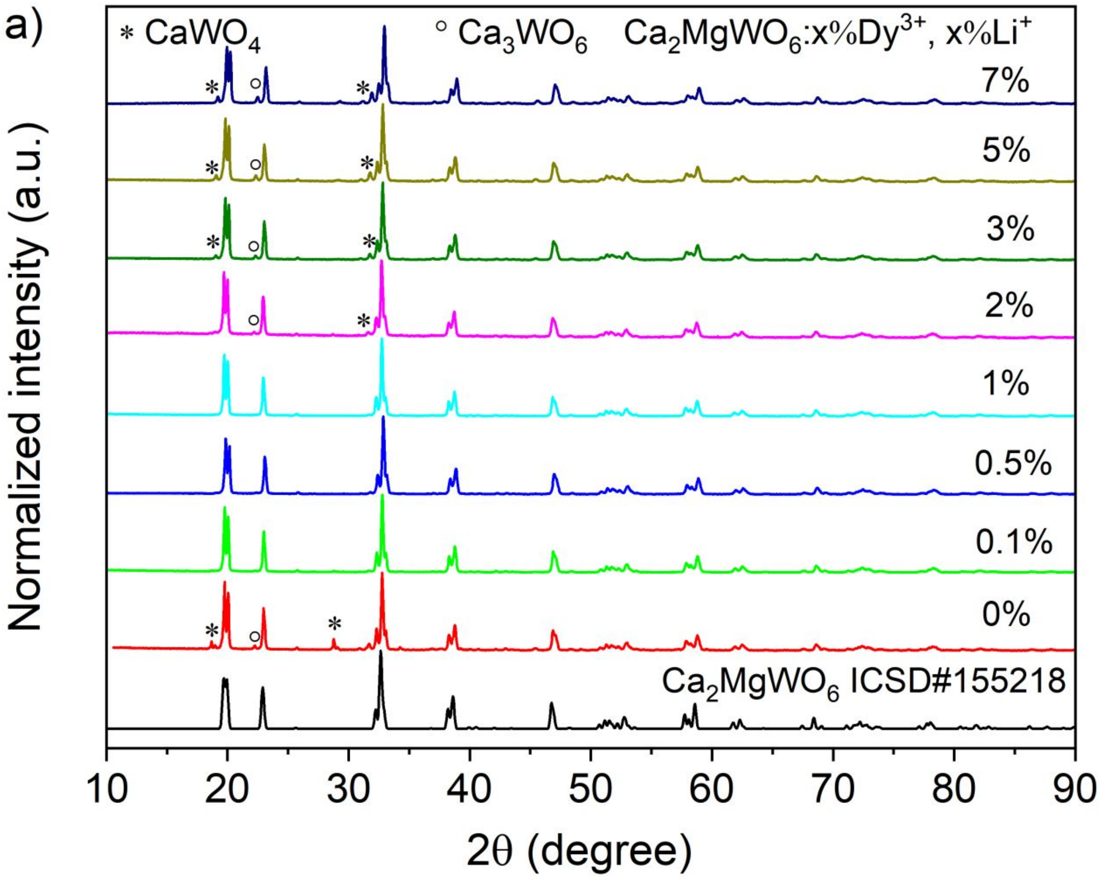
* CaWO4
○ Ca3WO6
Ca2MgWO6:x%Dy3+, x%Li+
7%
5%
3%
2%
1%
0.5%
0.1%
0%
Ca2MgWO6 ICSD#155218
Normalized intensity (a.u.)
2θ (degree)
10
20
30
40
50
60
70
80
90

b)

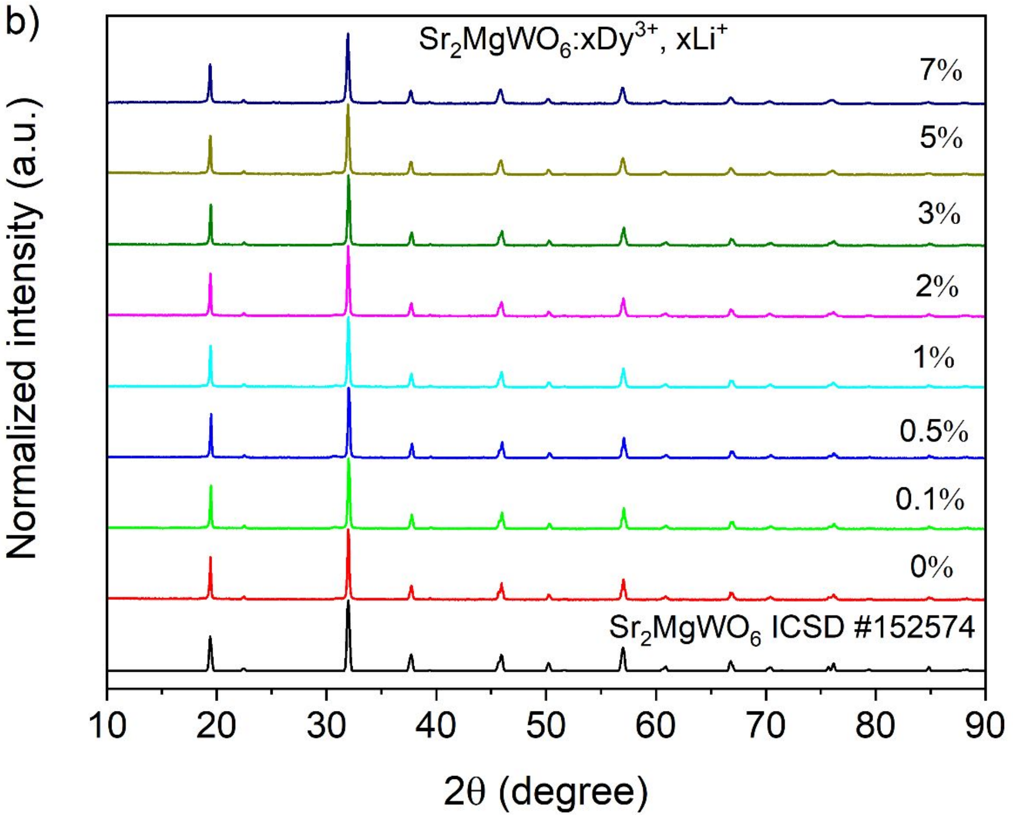
Sr2MgWO6:xDy3+, xLi+
7%
5%
3%
2%
1%
0.5%
0.1%
0%
Sr2MgWO6 ICSD #152574
Normalized intensity (a.u.)
2θ (degree)
10
20
30
40
50
60
70
80
90

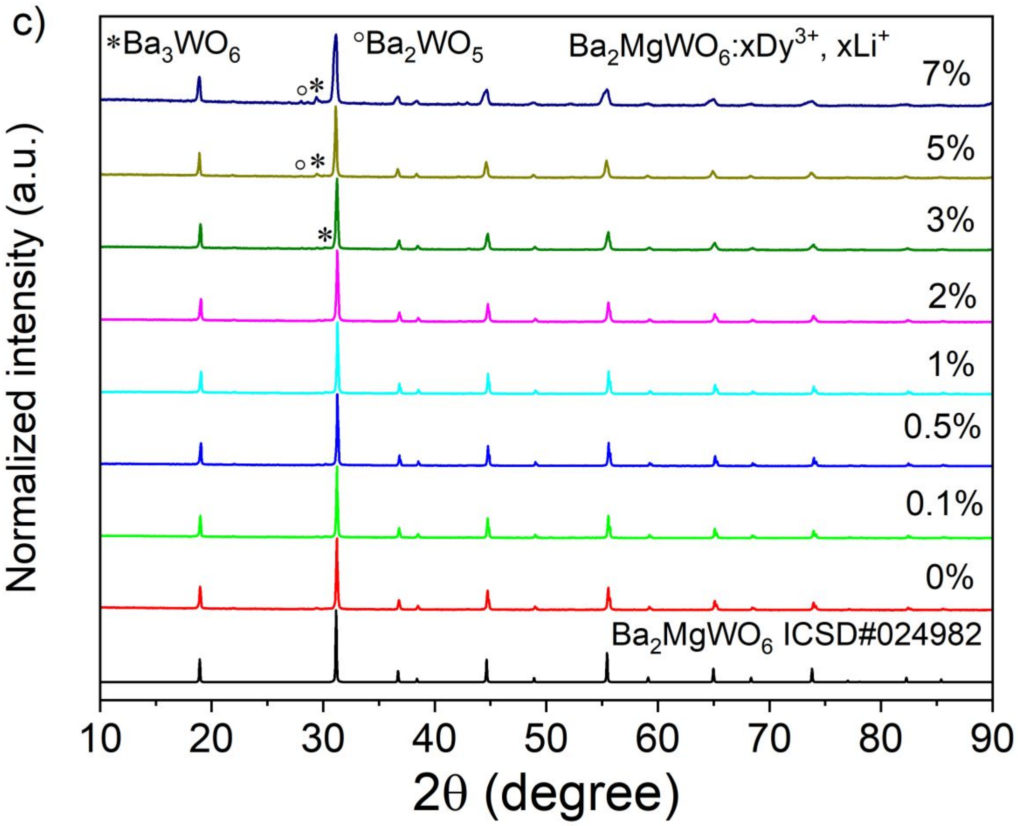


**Figure S1.** X-ray powder diffraction lines of $A_2Mg_{1-2x}WO_6$: xDy$^{3+}$, xLi$^{+}$ (x = 0, 0.001, 0.005, 0.01, 0.02, 0.03, 0.05, 0.07) where A = Ca (a), Sr (b), Ba (c)

**Table S1.** a) The refined lattice parameters, unit cell volumes, and uncertainties of the undoped and $A_2Mg_{1-2x}WO_6$: xDy$^{3+}$, xLi$^{+}$ (A = Ca, Sr, Ba; x = 0, 0.02)

| ***Chemical formula*** | ***$Ca_2MgWO_6$*** | ***$Sr_2MgWO_6$*** | ***$Ba_2MgWO_6$*** |
|---|---|---|---|
| ***Formula weight*** *(g/mol)* | *384.3* | *479.4* | *578.8* |
| ***Temperature*** | *Room temperature* | *Room temperature* | *Room temperature* |
| ***Pressure*** | *Atmospheric* | *Atmospheric* | *Atmospheric* |
| ***Crystal system*** | *monoclinic* | *tetragonal* | *cubic* |
| ***Space-group*** | *$P\ 2_1/n$ (#14)* | *I 4/m (#87)* | *$Fm\bar{3}m$ (#225)* |
| ***Z*** | *2* | *2* | *4* |

| *Sample* | *x = 0* | *x = 0.02* | *x = 0* | *x = 0.02* | *x = 0* | *x = 0.02* |
|---|---|---|---|---|---|---|
| ***a (Å)*** | *5.420(9)* | *5.427(6)* | *5.58(1)* | *5.584(2)* | *8.098(1)* | *8.10(7)* |
| ***b (Å)*** | *5.545(1)* | *5.547(3)* | *5.58(1)* | *5.584(2)* | | |
| ***c (Å)*** | *7.714(9)* | *7.725(6)* | *7.935(4)* | *7.942(1)* | | |
| *α (°)* | *90* | | *90* | | *90* | |
| *β (°)* | *89.91* | | *90* | | *90* | |
| *γ (°)* | *90* | | *90* | | *90* | |
| ***V (Å³)*** | *231.9(1)* | *232.6(1)* | *247.2(5)* | *247.6(6)* | *531.0(8)* | *532.8(9)* |
| ***χ2*** | *46* | *25.1* | *34.46* | *4.79* | *12.1* | *7.93* |
| ***$R_p$ (%)*** | *10* | *4.38* | *6.39* | *2.73* | *7.2* | *5.05* |
| ***$R_{wp}$ (%)*** | *17.9* | *8.55* | *11.2* | *3.80* | *10.3* | *7.07* |

**Table S1.** b) Crystallographic parameters from ICSD card no. 155218 of $Ca_2MgWO_6$, no. 152574 of $Sr_2MgWO_6$, and no. 024982 of $Ba_2MgWO_6$

| ***Chemical formula*** | ***$Ca_2MgWO_6$*** | ***$Sr_2MgWO_6$*** | ***$Ba_2MgWO_6$*** |
|---|---|---|---|
| ***Formula weight*** *(g/mol)* | *384.3* | *479.4* | *578.8* |
| ***Temperature*** | *473 K* | *298 K* | *Room temperature* |
| ***Pressure*** | *Atmospheric* | *Atmospheric* | *Atmospheric* |
| ***Crystal system*** | *monoclinic* | *tetragonal* | *cubic* |
| ***Space-group*** | *$P\ 2_1/n$ (#14)* | *I 4/m (#87)* | *$Fm\bar{3}m$ (#225)* |
| ***Z*** | *2* | *2* | *4* |
| ***a (Å)*** | *5.4415(2)* | *5.5876(1)* | *8.1120* |
| ***b (Å)*** | *5.5538(1)* | *5.5876(1)* | *8.1120* |
| ***c (Å)*** | *7.7413(2)* | *7.9490(3)* | *8.1120* |
| *α (°)* | *90* | *90* | *90* |
| *β (°)* | *89.93(1)* | *90* | *90* |
| *γ (°)* | *90* | *90* | *90* |
| ***V (Å³)*** | *233.95* | *248.18(1)* | *533.81* |

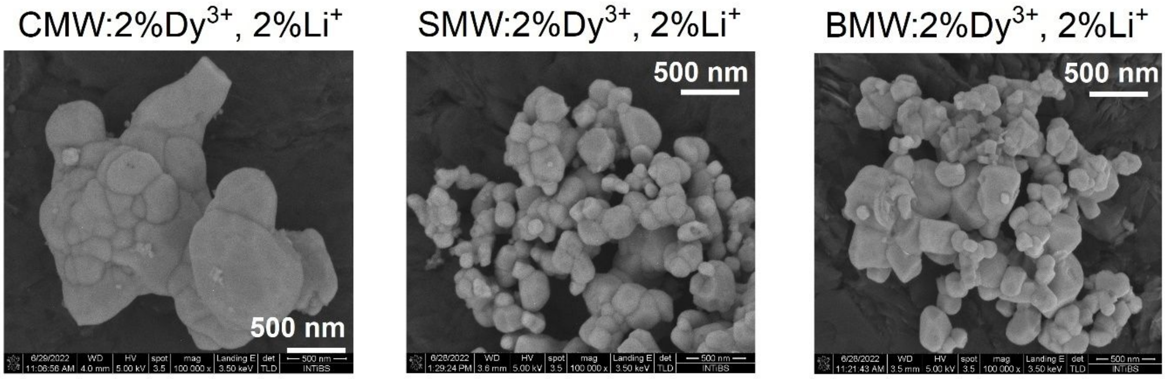


**Figure S2.** SEM images of $A_2MgWO_6$:2%$Dy^{3+}$, 2%$Li^+$ (A = Ca, Sr, Ba)

**Table S2.** The actual weight (%) of lithium in the prepared samples

| *Sample* | *Nominal weight % of lithium* | *Actual weight % of lithium* |
|---|---|---|
| $Ca_2Mg_{0.6}Dy_{0.2}Li_{0.2}WO_6$ | *0.34* | *0.173 ± 0.017* |
| $Sr_2Mg_{0.6}Dy_{0.2}Li_{0.2}WO_6$ | *0.28* | *0.136 ± 0.014* |
| $Ba_2Mg_{0.6}Dy_{0.2}Li_{0.2}WO_6$ | *0.23* | *0.109 ± 0.011* |

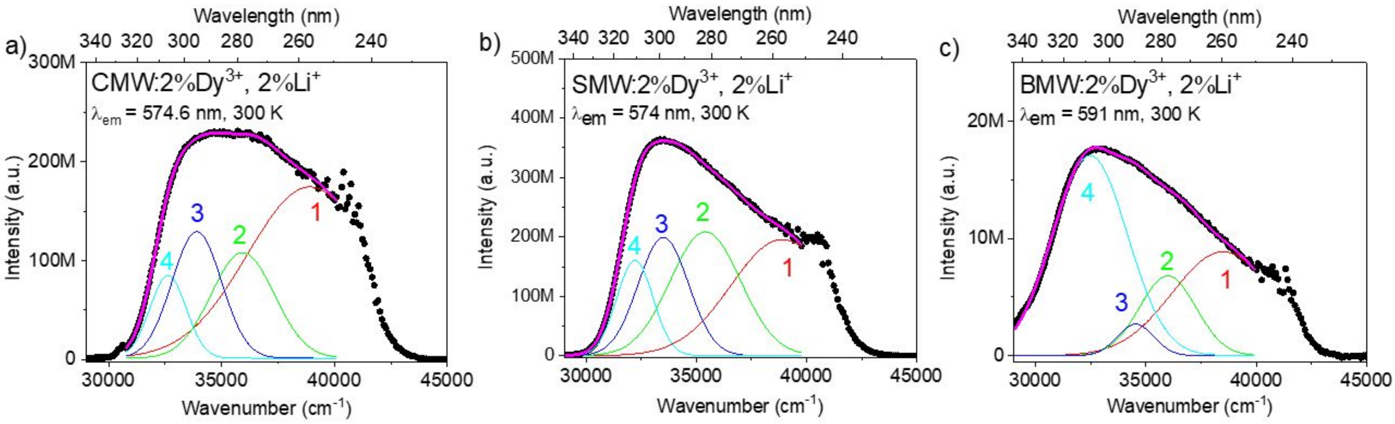


**Figure S3.** Gauss fitting of the 300 K excitation spectra of a) CMW: 2%$Dy^{3+}$, 2%$Li^+$; b) SMW: 2%$Dy^{3+}$, 2%$Li^+$; and c) BMW: 2%$Dy^{3+}$, 2%$Li^+$ monitored at 574.6, 574, and 591 nm, respectively. Please note that free ozon lamp emits from 230 nm.

**Table S3.** Gauss fitting peaks (in $cm^{-1}$) of the 300 K excitation spectra of $A_2MgWO_6$:2%$Dy^{3+}$, 2%$Li^+$ (A = Ca, Sr, Ba)

| Fitting peaks | peak 1 | peak 2 | peak 3 | peak 4 | Area_peak$_{1,2}$/Area_peak$_{3,4}$ |
|---|---|---|---|---|---|

| | | | | | |
|---|---|---|---|---|---|
| CMW: 2%$Dy^{3+}$, 2%$Li^{+}$ | 38910 | 35934 | 33900 | 32626 | 3 |
| SMW: 2%$Dy^{3+}$, 2%$Li^{+}$ | 38890 | 35403 | 33495 | 32202 | 2.2 |
| BMW: 2%$Dy^{3+}$, 2%$Li^{+}$ | 38492 | 35985 | 34531 | 32468 | 0.9 |

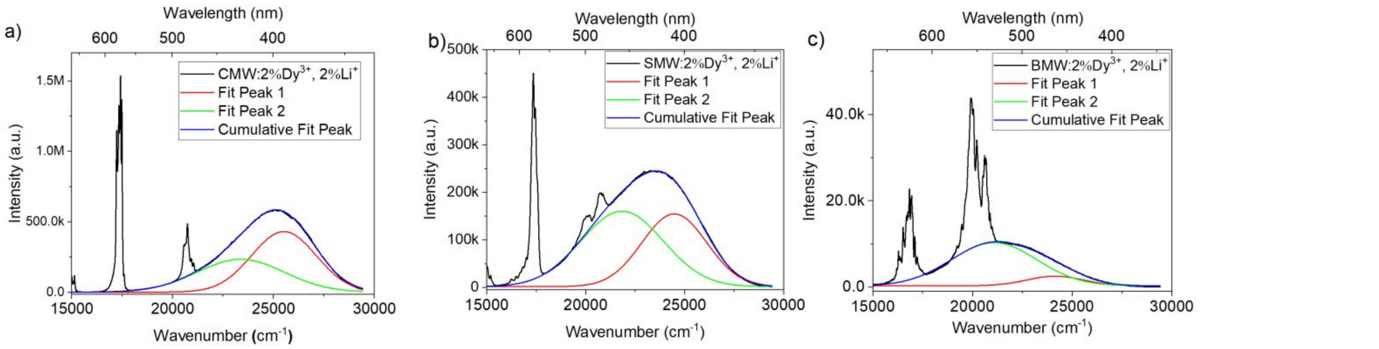


**Figure S4.** Gauss fitting of the 80 K emission spectra of a) CMW: 2%$Dy^{3+}$, 2%$Li^{+}$; b) SMW: 2%$Dy^{3+}$, 2%$Li^{+}$; and c) BMW: 2%$Dy^{3+}$, 2%$Li^{+}$ excited at 285 nm, 300 nm, and 306 nm, respectively

**Table S4.** Gauss fitting peaks (in $cm^{-1}$) of the 80 K emission spectra of $A_2MgWO_6$:2%$Dy^{3+}$, 2%$Li^{+}$ (A = Ca, Sr, Ba)

| Fitting peaks | peak 1 | peak 2 | Area_ $peak_1$/ Area_$peak_2$ |
|---|---|---|---|
| CMW: 2%$Dy^{3+}$, 2%$Li^{+}$ | 25518 | 23379 | 1.36 |
| SMW: 2%$Dy^{3+}$, 2%$Li^{+}$ | 24480 | 21842 | 0.76 |
| BMW: 2%$Dy^{3+}$, 2%$Li^{+}$ | 24147 | 21079 | 0.15 |

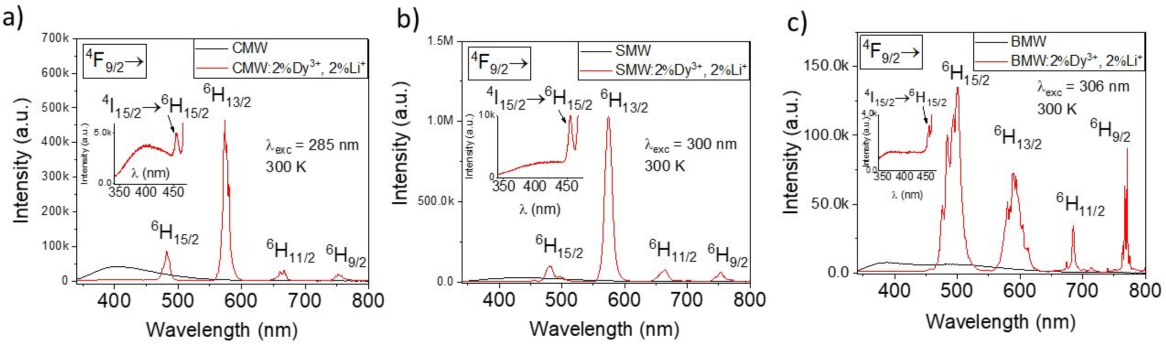


**Figure S5.** 300 K emission spectra of a) CMW and CMW: 2%$Dy^{3+}$, 2%$Li^{+}$; b) SMW and SMW: 2%$Dy^{3+}$, 2%$Li^{+}$; c) BMW and BMW: 2%$Dy^{3+}$, 2%$Li^{+}$ under 285, 300, and 306 nm excitation.

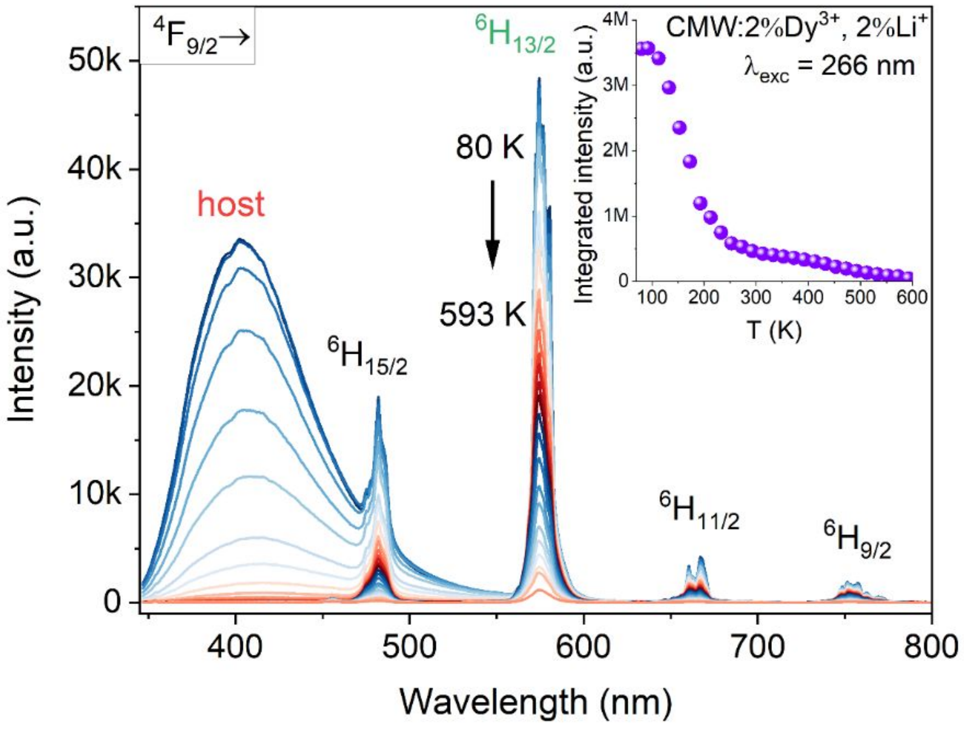


**Figure S6.** Temperature-dependent emission spectra of CMW: 2%$Dy^{3+}$, 2%$Li^{+}$

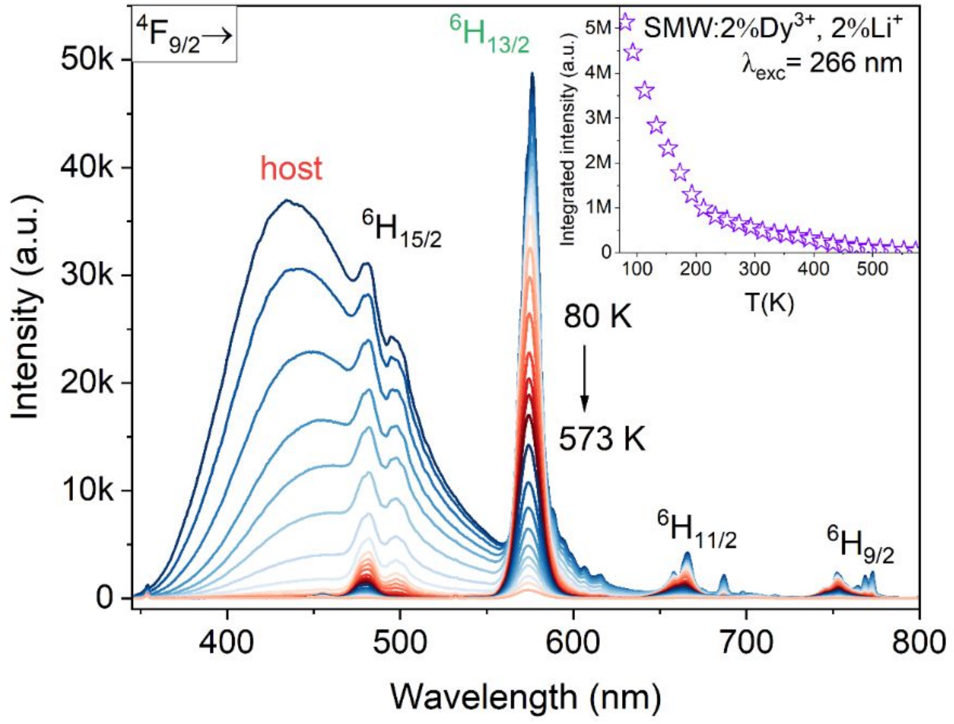


**Figure S7.** Temperature-dependent emission spectra of SMW: 2%$Dy^{3+}$, 2%$Li^{+}$

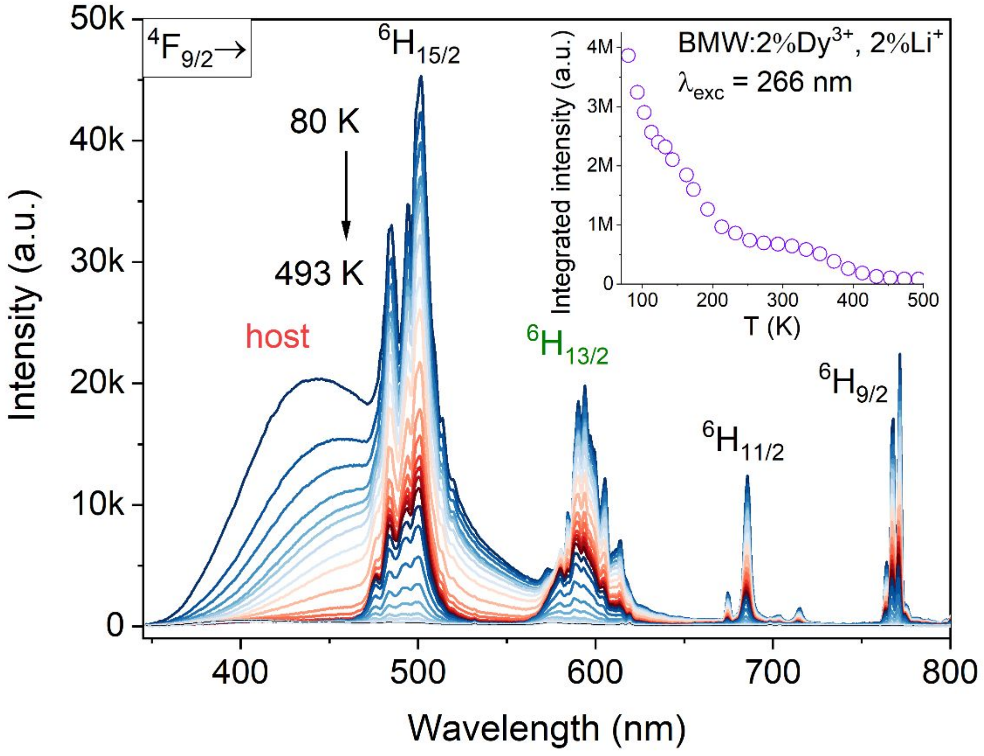


**Figure S8.** Temperature-dependent emission spectra of BMW: 2%$Dy^{3+}$, 2%$Li^+$

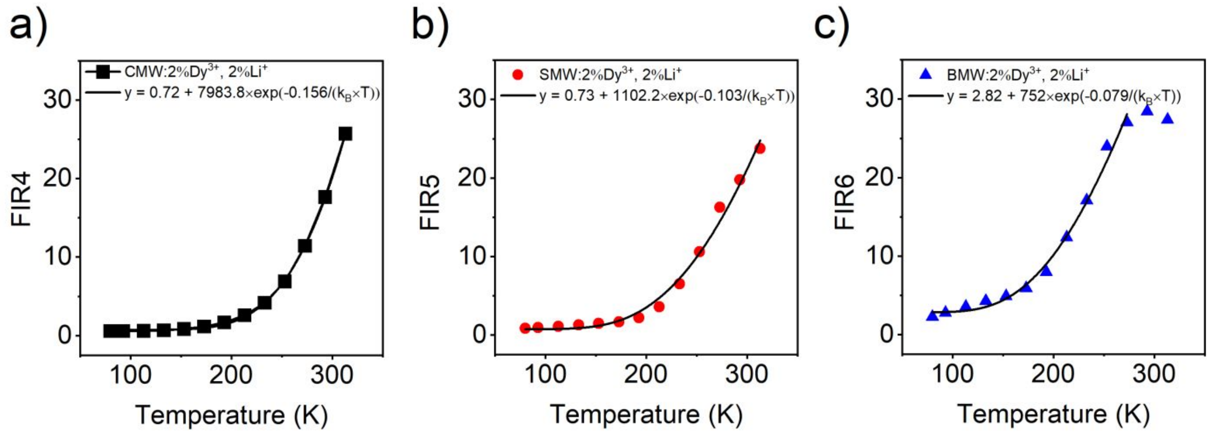


**Figure S9.** The relationship between the $FIR_{4,\ 5,\ 6}$ and temperature of $A_2MgWO_6$:2%$Dy^{3+}$, 2%$Li^+$ where A = Ca (a), Sr (b), Ba (c) from left to right

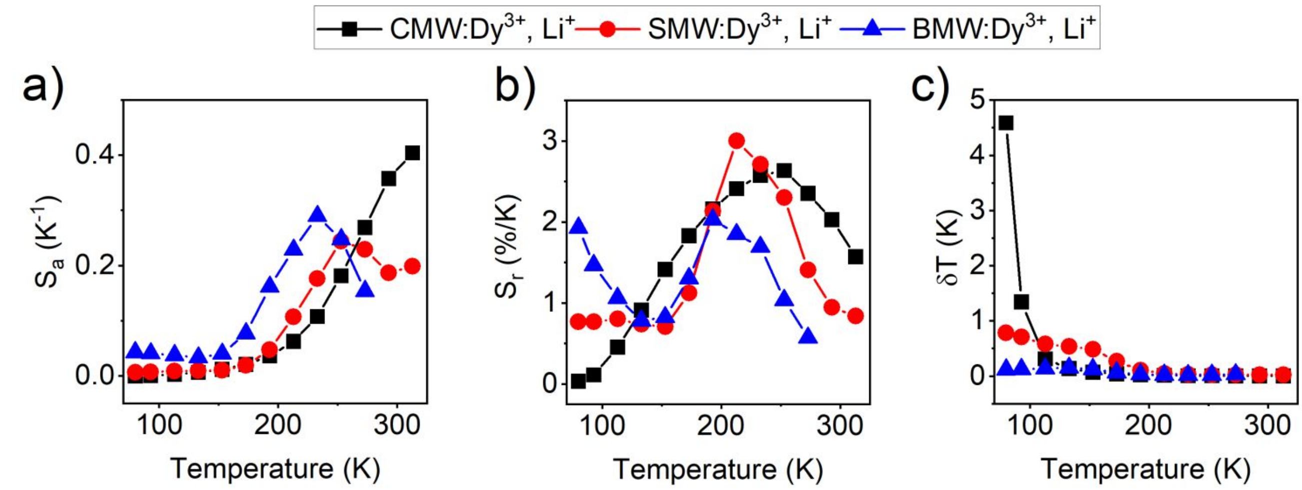


**Figure S10.** a) Absolute sensitivity $S_{a\_4,5,6}$ b) Relative sensitivity $S_{r\_4,5,6}$ c) Temperature uncertainty $\delta T_{4,5,6}$ versus temperature

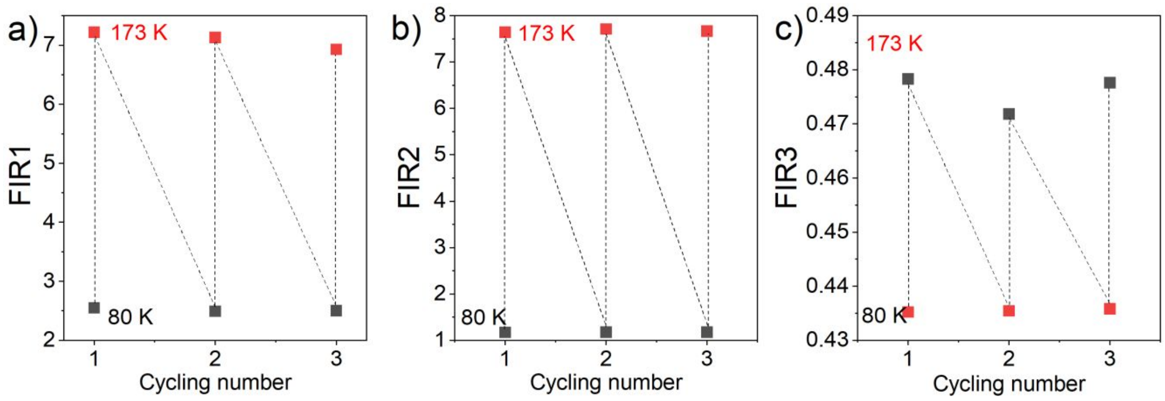


**Figure S11.** Repeatability of $FIR_{1,2,3}$ at 80 K and 173 K during the heating/cooling cycles of $A_2MgWO_6$:2%$Dy^{3+}$, 2%$Li^+$, A = Ca (a), Sr (b), Ba (c) from left to right

Repeatability R (%) was calculated via the following formula.

$$R = \left[1 - \frac{\max\left(|\Delta_m - \Delta_i|\right.}{\Delta_m}\right] \times 100\%$$

where $\Delta_m$ is the mean value of thermometric parameter ($\Delta$) at each temperature and $\Delta_i$ is the value of each specific measurement.
